# Supplementary material for: Association between nutritional status and dengue severity in Thai children and adolescents
Source: PLoS Negl Trop Dis. 2022 May 19;16(5):e0010398. doi: 10.1371/journal.pntd.0010398 (PMC9159591; doi:10.1371/journal.pntd.0010398)
Supplement: S1 Table — (DOCX) [file pntd.0010398.s001.docx]

**S1 Table.** Z score of BMI and dengue severity according to the 1997 WHO classification

| Nutritional status  Mean Z score (SD) | DHF  (n=59) | DF  (n=296) | P-value |
| --- | --- | --- | --- |
| Underweight (n=32) | -1.57 (0.29) | -1.68 (0.29) | 0.43 |
| Overweight (n=102) | 3.38 (1.98) | 2.88 (1.44) | 0.26 |
| Obese (n=57) | 5.0 (1.48) | 3.79 (1.27) | 0.25 |

 Abbreviations: BMI, body mass index; WHO, World Health Organization; SD, standard deviation; DHF, dengue hemorrhagic fever; n, number; DF, dengue fever; SD, standard deviation
