# Supplementary material for: Association between nutritional status and dengue severity in Thai children and adolescents
Source: PLoS Negl Trop Dis. 2022 May 19;16(5):e0010398. doi: 10.1371/journal.pntd.0010398 (PMC9159591; doi:10.1371/journal.pntd.0010398)
Supplement: S2 Table — (DOCX) [file pntd.0010398.s002.docx]

**S2 Table**. Z score of BMI and dengue severity according to the 2009 WHO classification

| Nutritional status  Mean Z score (SD) | Severe dengue  (n=76) | Non-severe dengue  (n=279) | P-value |
| --- | --- | --- | --- |
| Underweight (n=32) | -1.55 (0.25) | -1.7 (2.96) | 0.22 |
| Overweight (n=102) | 3.15 (1.9) | 2.91 (1.45) | 0.56 |
| Obese (n=57) | 4.79 (1.49) | 3.8 (1.28) | 0.05 |

 Abbreviations: BMI; body mass index; WHO, World Health Organization; SD; standard deviation; n, number
