# Supplementary material for: Association between nutritional status and dengue severity in Thai children and adolescents
Source: PLoS Negl Trop Dis. 2022 May 19;16(5):e0010398. doi: 10.1371/journal.pntd.0010398 (PMC9159591; doi:10.1371/journal.pntd.0010398)
Supplement: Attachment — Submitted filename: Responding to Reviewers-10-2-22.docx [file pntd.0010398.s003.docx]

**Responding to reviewer**

**Methods**

**Reviewer #1:** The study design is not fully appropriate for the stated objectives.

•	Suggest to compare OPD and IPD group to see any different in their nutritional status or not and analyze each group separately?

Replied: Thank you for your suggestion. Then we add OPD/IPD and nutritional status information in result. There was no difference of nutrition distribution between OPD/IPD groups, as shown in table 1.

•	Suggest to add more detail of clinical and hospital management, eg. complications (hepatitis, AKI, bleeding,…), Dextran, blood transfusion, Length of stay in the hospital

Replied: The information of major organ involvement, management length of stay were added in the Table 1 and 2.

**Reviewer #2:** The authors did not describe a designed retrospective chart review with the classification of dengue severity based on both the 1997 and 2009 Dengue WHO guidelines.

- They did not show how they sample, record, and review the dengue cases as well as confirm the classifications.

Replied: we add more study design details including type of study, how to evaluated the dengue classification in material and method.

Briefly:

- All available medical records of paediatric patients who diagnosed dengue infection were retrieved from both outpatient and inpatient departments from 1 January 2017 to 31 December 2019.

- information of nutritional status includes age, weight and height and dengue severity includes haematocrit on defervescent date and its baseline, vital signs and bleeding information etc.

- For in case of dengue classification disagreement between final diagnosis in medical record and investigator’s judgment, it would be re-justified by the group of independent paediatricians.

- Furthermore, they should clarify why they included the dengue cases with underlying diseases in the study.

Replied: Thank you for your comment. We realized that the underlying may influence to dengue outcome. However, for this study, all underlying condition were mild and the not difference of distribution of presenting of underlying disease and dengue severity. Therefore, we would like to keep this information and mention the discussion part as “ In this study, 52 (14.6%) patients who had underlying diseases were included in analysis because they were mild conditions and had no difference in distribution among dengue severity.”

**Reviewer #3:** 0-17 years is a *huge* age range. I would strongly suggest to do some sort of adjustment or stratification across age groups. It is possible that if an association exists, it is different in the very young vs. adolescents.

o	I would also suggest adjusting for sex of the child in addition.

o	Is there a reason why 11-17y are together in one group? This is almost 80% of the sample, could split this group.

Replied: Thank you for your suggestion. Therefore, we re-stratified the age to be 0-2, 3-5,6-10, 11-14 and 15-17 years. And we demonstrate the distribution in Table1 and compare dengue severity in Table 2 and 3. it was found the significant association of older age and severe dengue /DHF which may relate to secondary dengue infection. Therefore, we note this finding in discussion as “The result of study also demonstrated higher proportion of severe dengue and DHF in the older children which may relate to secondary dengue infection.”. We also tried subgroup analysis by age <15 and > 15 yr, However, there were no statistical significances between dengue severity and nutrition status. Therefore, we did not add this information in our result.

For the sex, there was no difference of dengue severity and sex as shown in Table 2 and 3.

**Reviewer #1:** The results are not clearly and completely presented.

Figure 1 – prefer to present as tables with p-values

Table 1

•	Suggest to add 2 more columns of OPD (n = 113), IPD (n = 242) group in Table 1.

•	Underlying diseases may be excluded because this contributes to more severe disease and complication? If underlying disease remained in the manuscript, please specify in each category of the nutritional status.

Replied: Figure 1 was replaced by Table 2 and 3. Table 1 was modified by added OPD/IPD columns. All underlying condition were mild and the presenting of underlying condition was compared among severity of dengue in Table 2 and 3. There were no differences.

Therefore, we would like to keep this data.

•	Parasite infection, please specify. If only minor parasitic infection without fever or systemic signs and symptoms, they may be omitted in underlying diseases?

Replied: Thank you for your suggestion. All parasitic infections were mild and accidentally found. We omitted this information and adjust number of case with underlying disease.

Table 2 & 3

•	Suggest to add another 2 tables of % of DSS, DHF, DF and Severe, non-severe dengue in and please clarify category of severe dengue, whether shock, respiratory distress, bleeding or organ (s) failure.

Replied: Table 2 and 3 were modified according to suggestion.

•	Separate OPD and IPD group will be presented as in Table 2 &3.

Replied: Demographic data was present with OPD/IPD column in Table 1 and we added row of IPD group in Table 2 and 3.

**Results**

•	Page 5 – explanation of Figure 1, please add % of obese patients in both WHO 1997 and 2009 classifications. In WHO 1997, please add 5 of DSS and DHF separately. In WHO 2009, please add detail category of severe dengue, i.e. shock, respiratory distress, bleeding or organ (s) involvement.

Replied: They were added in table 2 and 3

•	Page 7, first paragraph: please re-calculate % of normal weight (62.3%) and overweight/obese (28.7%). The number for normal weight = 164, overweight = 102 and overweight = 57 and the total number = 355 patients.

Replied: We do confirm that the number of underweight, normal weight and overweight were 32(9.0%), 221 (62.3%) and 102 (28.7%) in total of 355. Obese (57/355 =16%) was a subset of overweight.

**Reviewer #2:** - For Figure 1, instead of the bar chart, the data should be represented in Table to more clarify and reduce repeated picture.

Replied: It was replaced with Table 2 and 3.

- The clinical characteristics of pediatric patients should be represented and shown the differences among groups.

Replied: They were added in Table 1.

**Reviewer #3:** Results

Add an explanation of why cases were excluded (574 records were screened and 355 met inclusion/exclusion criteria) – could add information that XX were over 18y, xx did not have nutritional status, etc.

Replied: This information was added in the first part of result section as “355 cases were included and 219 cases were excluded as age were greater than 17 years (112 cases), non-Thai (37 cases), unavailable result of laboratory confirmed dengue infection (44 cases), and unavailable clinical data (26 cases).”

•	Table 2 and Table 3 (Now are table 4 and 5)

- Incorrect labelling, the first column is not “n(%)” – that is rather for the 2nd and 3rd columns (and an accurate heading should be put for the first column information)

Replied: It was corrected.

o	Add what the reference group is (tables should be self-contained) I corrected it

- Define all acronyms used in the table (tables should be self-contained)

Replied: It was corrected.

**Conclusions**

**Reviewer #1:** The conclusion will depend on the suggested study design.

**Reviewer #2:** The authors may discuss why to choose the BMI-for-age Z-score as an indicator based on WHO nutritional classification, instead of WHZ based on Thai Growth chart.

Replied: More explanation in discussion was added as “Z-scores for the BMI in each patient which may provide a fine details of nutrition status,” in discussion part.

**Reviewer #3:**

**Discussion**

- It seems like the authors looked for something and did not find it.. but it is difficult as the reader to piece together why they were looking for it in the first place. From this paper, I was not convinced that this was worth looking for or what they really added to the literature on this topic. I would have expected an explanation of the biological plausibility, rationale, and implications for clinical work / policy that makes this topic worth examining.

Replied Thank you for your suggestion. The biological plausibility was added in introduction as “Significantly, nutrition was matter to immune functions which it could influence on genomics and metabolisms, and the role of adipose tissue in overweight could stimulate more inflammatory mediators which leading to increase capillary permeability and plasma leakage. However, whether nutritional status is really the risk of severe dengue, there might need more further evidences.”

o	A systematic review was cited so it seems this is worth examining but there was little to no justification of why this is helpful to look into for the reader of this paper. Add this so that the reader understands the biological plausibility and relevance, in particular, of an association between overweight/obesity and mild plasma leakage.

- Weight was taken on the first day of hospitalization. Add a point to the discussion of the potential bias that this may have (if weight loss already occurred during the disease progression prior to hospital admittance)? Or why this should *not* be an issue.

Replied: The sentence “the weight on the first day of visit may not reflect the ideal underlying weight due to loss of appetite, however it was the practical underlying weight” in discussion.

**Editorial and Data Presentation Modifications**

**Reviewer #1:** As in the above suggestion

**Reviewer #2:** (No Response)

**Reviewer #3:** (No Response)

**Summary and General Comments**

**Reviewer #1:** To identify the association between nutritional status and dengue severity need the same population, i.e. only the admitted patients. OPD patients, about 1/3 of the study population in this study tend to have milder illness that may interfere with the results of the study.

Replied: Thank you for your comment

**Reviewer #2:** Please specify how the data was extracted and by whom as well as reviewed and confirmed in the classifications.

Please clearly state inclusion/exclusion criteria with a brief rationale.

Please reveal the clinical characteristics of patients.

Replied: We provided more information in method and result sections.

**Reviewer #3:** What was the distribution of nutritional status of the general population that you were looking at (children in urban Thailand/Bangkok)?

o	To do this you could look at distribution of all at the clinic / or a comparison group (with another, clearly unrelated condition – or those children coming for wellness checks and therefore presumably healthy?)

o	Published survey data from a large survey done in Bangkok recently (or at least in urban areas of Thailand?)

o	For example, this national survey has much lower %s of overweight and obesity:

	National survey of overweight/obesity: https://www.ncbi.nlm.nih.gov/pmc/articles/PMC5824639/

o	This more recent article, however, mentions a survey with numbers more even higher than what was found in this study (36% obesity in high school students and 19.6% in primary-school-age children). But they don’t cite the specific survey. (<https://www.bumrungrad.com/en/health-blog/october-2019/child-obesity-becoming-a-growing-problem>)

o	Overall, my point is that these numbers need some context. How is this population of children different from this age group living in Bangkok? Even without collecting/calculating anything further you can add this information. In particular, among those 11-17y as that makes up the vast majority of the sample.

Replied: Thank you for your suggestion So we added the distribution of nutritional status in the introduction part as “Both under and over nutrition are health issue. In Thailand, overweight had been appeared an emerging disorder. The prevalence of obesity from 3 to 18 years old of children and adult were 9.1% and 6.5% respectively [5]. However, the rate could be changed from urban and rural population.”

**Introduction**

o	The rationale why you are looking at this association in the first place remains unclear. Justify why it is thought that underweight and, in particular, overweight may be associated with more severe dengue outcomes.

o	Be more explicit about what this will mean for clinicians and researchers – if you find there is an association.

o	I would call them children and adolescents. It seems a bit misleading to say just children when almost 80% are 11-17 years.

Replied: Thank you for your mention. We add more information in introduction and modified the title to “Association between nutritional status and dengue severity in Thai children and adolescents”
